# Nutritional and physical properties of organic Beauregard sweet potato [*Ipomoea batatas* (L.)] as influenced by broiler litter application rate

**DOI:** 10.1002/fsn3.108

**Published:** 2014-04-09

**Authors:** Peter N Gichuhi, Kokoasse Kpomblekou-A, Adelia C Bovell-Benjamin

**Affiliations:** 1 Department of Food and Nutritional Sciences, Tuskegee UniversityTuskegee, Alabama, 36088; 2 Department of Agricultural and Environmental Sciences, Tuskegee UniversityTuskegee, Alabama, 36088

**Keywords:** Beauregard sweet potato, broiler litter, manure application rate, nutrition

## Abstract

Organic farming has been on an upward trend in recent years. However, the manures used like broiler litter have variable nutrient content, making it important to establish optimal application rate, for maximum crop yield and quality. Additionally, some states like Alabama restricts the amount of broiler litter to control excessive nutrients accumulation which can lead to surface and ground water contamination. The current study evaluated the effect of broiler litter at rates 0, 0.5, 1, 2, and 3 t ha^−1^ (treatments T_0_, T_0.5_, T_1_, T_2_, and T_3_), on the nutritional and physical properties of Beauregard sweet potato. Analyses were performed to determine moisture, ash, fiber, vitamin C, and *β*-carotene contents using oven, muffler furnace, dye, and spectrophotometric methods; texture; and color using compressive strength and *L*, *a*, *b* system, respectively. Ash content of the samples ranged from 0.9% to 1.4% with a very strong positive linear correlation (*r* = 0.9) to the broiler litter rate. However, vitamin C had a quadratic relationship with the broiler litter rate with a peaking at T_0.5_ (15.5 mg/100 g). The yellow color (*b*-value) also had a strong linear relationship with the broiler litter rate (*r* = 0.86). However, the other measures showed moderate, weak, or negligible correlations to the broiler litter level. T_0.5_ had the highest *β*-carotene (262.0 *μ*g/g), dry matter contents and had the most firm (0.040 kN) sweet potatoes with the deepest orange color (*L* = 60.7). Based on the study's findings, 0.5 t ha^−1^ appeared to be appropriate level of broiler litter, which is consistent with Alabama's law and is also advantageous in terms of low cost of farming practices and water pollution reduction.

## Introduction

Organic farming has received a lot of attention in the past few decades. From the 1980s, organic farming has had a steady growth, and has recently increased tremendously worldwide (Stolze and Lampkin 2009). For labeling a product as organic, farmers have to meet some standard practices during the growth process. For example, according to the USDA National Organic Program, one of the standard practice is the use of organic fertilizer, which is a soil amendment derived from natural resources that guarantees at least the minimum percentage of N, P, and K. Hence, the soil is fertilized through tillage/cultivation, crop rotation, cover crop, and use of animal or plant waste material (USDA/National Organic Program 2013). However, when using animal waste as a fertilizer, it is essential to establish an optimum application rate, for a particular crop. The reason for establishing the manure optimal rate is to avoid underfertilization or overfertilization, which can adversely affect the crop of interest. As reported by Bary et al. (2000), application of too little manure could lead to inadequate growth of crop. On the other hand, applying too much manure may reduce the quality of crop and can also lead to surface and ground water contamination. For example, some mineral nutrients like Cu and Cl are toxic to plants when applied in very high concentration, beyond requirement of a particular crop (O'Sullivan et al. 1997). In addition, excessive nutrients in the soil can cause degradation of both surface and ground water through surface runoff and leaching, which is a growing environmental concern. For example, Gilfillen et al. (2010) observed that application of broiler litter to meet the N need of orchardgrass in a 4-year study resulted in a three-, seven-, and fivefold soil accumulation of P, Cu, and Zn, respectively. For such reason, Alabama Cooperative Extension Program recommends application of broiler litter at a rate of 4.5 t ha^−1^ year^−1^ and emphasizes on residual soil N consideration when applying nutrients in subsequent seasons Mitchell and Donald (1999).

Adequate supply of plant nutrients is important for healthy growth of crops as they are the chemical elements which form the plant tissues (O'Sullivan et al. 1997). For example, N is the key element in protein synthesis. Although the most common plant elements (C, O, and H) come from water and air, the other, macronutrients (N, K, P, Ca, Mg, and S) and micronutrients (Fe, Cl, B, Mn, Zn, Cu, and Mo), come from the soil. When deficient in the soil, these mineral nutrients may be supplemented as inorganic or organic fertilizer to achieve healthy crop growth. However, mineral nutrients like boron, chlorine, manganese, and copper are toxic to plants if applied in high concentrations. In addition, Al and Na, which are not essential to sweet potato, can also cause toxicity to the crop (O'Sullivan et al. 1997). It is, therefore, important to apply the right amount of the soil fertilizing material. Sometimes the optimal amount is not straight forward, especially when dealing with organic manure. Organic manures have slow release and variable nutrients composition depending on the source, as opposed to the chemical fertilizers, which have readily available nutrients and of known concentration.

According to Kingery et al. (1994), extensive use of broiler litter, can cause accumulation of components like organic C, total N, P, K, Ca, Mg, Cu, Zn, and NO_3_^−^ and also increase in the soil pH. However, element like Cu is toxic to plant at high concentration and elevated soil pH can reduce the solubility of nutrients like P, Fe, Mn, Zn, and Cu (O'Sullivan et al. 1997). In addition, although NO_3_^−^ is one of the major source of N, high concentration in the soil can lead to its accumulation in plants (Nesic et al. 2008), which can cause growth retardation. Chen et al. (2004) observed the effect of application of N in form of KNO_3_ in potted vegetables at levels, 0.00, 0.15, 0.30, 0.45, and 0.60 g N per 5 kg soil. The results from the study indicated that the 0.30 g N/5 kg level had the maximum yield and the 0.00 g N/5 kg level had the minimum yield. This means that the 0.30 g N/5 kg was the optimum application rate of the KNO_3_ fertilizer. Excessive accumulation of NO_3_^−^ in plant can also be harmful when consumed by animals. Plants normally respond to nutrients imbalance by decrease in growth rate and development. In addition, symptoms of the imbalance may only be visible on the plant when at severe levels, for example chlorosis, the reduction in the green color (O'Sullivan et al. 1997). However, some anomalies may not be visually notable on the crop or harvest if the nutrients aberration is not at a critical level. Therefore, nutritional analysis is important in determining the qualitative status of crop under different treatments. In addition, most nutritional studies compare organic versus the conventional crops, but not the effect of manure application rate in the organic farming. In the current study, the nutritional and physical properties of organically grown Beauregard sweet potato were evaluated to determine the effect of application of broiler litter at rates of 0, 0.5, 1, 2, and 3 tons per hectare (t ha^−1^) in meeting the application rate recommended by Alabama Cooperative Extension Program.

## Materials and Methods

### Samples and sample preparation

Beauregard sweet potatoes were organically grown in the Department of Agriculture, Tuskegee University, with broiler litter at rates of 0, 0.5, 1, 2, and 3 t ha^−1^, reported as treatments T_0_, T_0.5_, T_1_, T_2_, and T_3_, respectively. Selected properties of the broiler litter composition are reported in Table 1. Each treatment was grown in randomly distributed rows with four blocks (6 m × 6 m) each. The rows were 2 m, and the blocks 4 m apart. The maximum broiler litter rate of 3 t ha^−1^ tested in our study was established from previous field research experiments conducted at Tuskegee University, which showed that sweet potato storage root yield and quality tend to decline above broiler litter rates of 3 t ha^−1^ when preceded by cover crops (crimson clover or black oat) in organic farming systems (K. Kpomblekou-A, pers. comm., 2014). The application rate was also consistent with the recommendation of the Alabama Cooperative Extension Program broiler litter application rate of 4.5 t ha^−1^ year^−1^ to prevent the potential of ground and surface water pollution by excess nutrients.

**Table 1:** Selected properties of the broiler litter used

			Inorganic N (g kg^−1^)	
			
pH	Organic C (g kg^−1^)	Total N (g kg^−1^)	NH_4_^+^-N	NO_3_^−^-N + NO_2_^−^-N
8.5	290	46	3.03	1.57

After harvest, the sweet potatoes were delivered to the Department of Food and Nutritional Sciences, Tuskegee University, for the nutritional analysis study. Sweet potato from the four different blocks of each treatment were mixed together and then randomly sampled for analysis. The sampled roots were washed, blot dried with paper towel, peeled, and finely diced together for subsequent analyses. All the analyses were done in triplicate.

### Moisture analysis

Sweet potato samples (5 g) were weighed into predried, preweighed aluminum pans and heated in a conventional oven overnight at 105°C (Bradley 1998). The pans, plus dry sample, were then cooled in a desiccator and reweighed. Moisture content was calculated as percent weight loss of a sample to the initial weight.

### Ash analysis

Samples (5 g) were weighed into predried crucibles, placed in a muffler furnace, and heated at 550°C for 12 h. The crucibles, plus ash, were then cooled in a desiccator and reweighed. Ash content was calculated as the percent of ash weight to the initial sample weight.

### Crude fiber analysis

An Ankom2000 Fiber Analyzer (Macedon, NY) was used to determine the fiber content of the sweet potatoes. As per the manufacturer's instructions, samples (1 g) were weighed into preweighed Ankom filter bags and heat sealed. The filter bags were placed on the trays of the bag suspender, inserted into the vessel of the fiber analyzer, and subjected to acid followed by base digestions, with hot water rinses between runs. After the acid/base digestions, the bags were removed from the analyzer, placed in a beaker, and the excess water gently squeezed out. The bags were then soaked in acetone for 5 min, air-dried on a wire screen, and then dried in oven for 2 h at 102°C. The dry bags, with digested samples, were reweighed, placed in preweighed crucibles, and heated in a muffler furnace at 600°C for 2 h, to remove the remaining organic matter (fiber). The crucibles were then cooled in a desiccator and reweighed to determine the ash (crude minerals) proportion, and consequently the fiber content. Percent of the proportion of the fiber to the initial sample weight was calculated to determine its content.

### Vitamin C analysis

Vitamin C (ascorbic acid) was determined using the 2,6-dichlorophenol indophenol titration method (Eitenmiller et al. 1998). Samples (2 g) were ground with mortar/pestle and homogenized with 10 mL of metaphosphoric/acetic acid solution. For each sample, the extract was vacuum filtered using a Buchner funnel and the residue reextracted two more times. The residue was then poured on the Buchner funnel and rinsed with about 15 mL of the extraction solution, until the filtrate was clear. The filtrates were pooled into a 50 mL volumetric flask and filled to the mark with the extraction solution. Three aliquots (10 mL) of the extract were pipetted into Erlenmeyer flasks and titrated with the indophenol dye to a faint pink endpoint. Vitamin C content was determined by comparing the volume of the dye used to titrate a sample to the volume of the dye used to titrate ascorbic acid standard of known concentration.

### *β*-Carotene analysis

Hexane:acetone (7:3) mixture was used to extract *β*-carotene from the sweet potato samples. Each sample (1 g) was extracted with 25 mL of the hexane–acetone mixture and then vacuum filtered, rinsing the sample residue with 15 mL of extraction solvent until the filtrate was clear. The filtrate was then transferred into a separating funnel, washed with 50 mL of distilled water, allowed to settle and the water portion discarded. The extract was then dried with 10 g of anhydrous Na_2_SO_4_, transferred into a 50-mL volumetric flask and filled up to the mark with the extraction mixture. The extract was then transferred into cuvettes and absorbance read at 450 nm using a Shamadzu U-1201 UV-Vis spectrophotometer (Shamadzu, Jiangsu, China). A calibration curve generated using *β*-carotene standard was used to determine the total *β*-carotene content.

### Texture analysis

Sweet potato samples were prepared for texture analysis by cutting cylindrical pieces, longitudinally through the roots, using a cork borer with diameter 6.5 mm. Texture was then determined using a Mini 44 Instron Machine (Instron, Canton, MA). The maximum force (kN) required to break a cylindrical piece of sweet potato with the crosshead from a height of 30 mm at a speed of 10 mm/min was determined as the firmness of the sweet potato.

### Color analysis

The peeled, finely diced sweet potato samples were filled into petri dishes (plastic plates) and covered. Color was then measured from three different positions on the plate cover, using a handheld Minolta Chroma Meter (Minolta, Osaka, Japan) and the measurements reported using the *L*, *a*, *b* color system.

### Statistical analysis

The triplicates among treatments were analyzed using the Analysis of variance (ANOVA) to determine whether there were differences among the means and Fisher's LSD at *P* < 0.05 to determine where the differences lied. Pearson's correlation was used to measure the strength of the relationships between broiler litter and the nutrition/physical measures.

## Results and Discussion

### Moisture content

The moisture content of the sweet potatoes in the current study ranged from 77.0% for T_0.5_ to 78.6% for T_2_ (Table 1). This range translates to dry matter content of about 21.4–23.0%, which is within the range of findings in some past studies. In a study of nine orange-fleshed and three cream-fleshed sweet potatoes grown in four agrogeographical sites, Laurie et al. (2012) found the dry matter content range to be 15.5–34.9%. Specifically, the Beauregard variety had dry matter content ranging from 17.3% to 25.1%. In another study which involved 49 sweet potato varieties, Oboh et al. (1989) found the dry matter content of the roots to be between 17.82% and 38.18%. In the current study, the broiler litter application rate did not seem to have much effect on the moisture content of the sweet potatoes, although the T_0.5_ sweet potatoes had slightly less moisture than the control, T_0_ (Table 2). The other three treatments had moisture contents which were not statistically (*P* < 0.05) different to the control. The moisture contents of the different sweet potato treatments showed a moderate positive linear relationship (*r* = 0.42) with the level of the broiler litter applied (Table 4). However, Nedunchezhiyan et al. (2010) observed that sweet potato grown without fertilizer had less dry matter content than the ones grown with fertilizer and Jarvan and Edesi (2009) also observed that potato dry matter content increased with addition of manure or mineral fertilizer. These past studies may have shown contrasting results to the current study possibly because of large difference in application rates. For instance, Jarvan and Edesi (2009) compared zero and 60 t ha^−1^ of cattle manure unlike in the current study which had a narrow range of 0–3 t ha^−1^ broiler litter.

**Table 2:** Moisture, fiber, and *β*-carotene contents of the sweet potato under the different treatments

Treament	Moisture (%)	Fiber (%)	p-Carotene (*μ*g/g)
T_0_	77.92 ± 0.14^ab^	0.95 ± 0.02^ab^	178.0 ± 28.5^C^
T_0.5_	76.98 ± 0.34^c^	0.89 ± O.06^b^	263.0 ± 18.7^a^
T_1_	77.19 ± 1.01^bc^	0.95 ± 0.05^ab^	139.0 ± 40.0^C^
T_2_	78.58 ± 0.15^a^	0.91 ± 0.02^ab^	191.2 ± 9.9^bc^
T_3_	77.84 ± 0.25^ab^	0.99 ± 0.05^a^	238.3 ± 25.3^ab^

Values on the same column with similar letters are not significantly different (*P* < 0.05). T_0_, T_0.5_, T_1_, T_2_, T_3_ refer to treatments and the subscripts represent tons of broiler litter applied.

**Table 3:** Texture of the sweet potatoes grown with the different amount of broiler litter manure

	Treatment				
	
	T_0_	T_0.5_	T_1_	T_2_	T_3_
Firmness (kN)	0.033 ± 0.006^b^	0.040 ± 0.004^a^	0.037 ± 0.004^ab^	0.034 ± 0.001^ab^	0.035 ± 0.003^ab^

Values on the same row with similar letters are not significantly different (*P* < 0.05). T_0_, T_0.5_, T_1_, T_2_, T_3_ refer to treatments and the subscripts represent tons of broiler litter applied.

**Table 4:** Linear correlation between sweet potato measure and the rate (t ha^−1^)

							Color		
							
Analysis	Moisture	Ash	Fiber	Texture	Vitamin C	*β*-Carotene	*L*-value	*a*-value	*b*-value
Correlation coefficient (*r*)	0.4230	0.9019	0.4686	−0.2020	−0.1833	0.2338	0.0637	0.1579	0.8563

### Ash content

**Figure 1:**
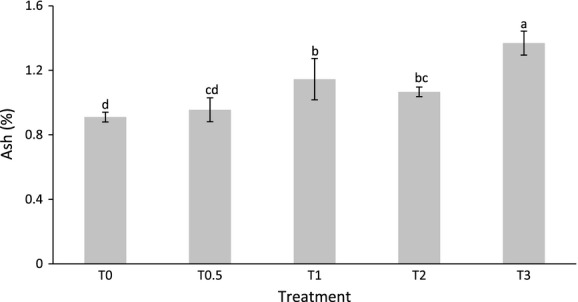
Ash content of the sweet potatoes growth with different amount of broiler litter.

Ash content of the sweet potato ranged from 0.91% for T_0_ to 1.37% for T_3_. Ash content of the sweet potatoes grown without broiler litter was significantly (*P* < 0.05) lower than that of the sweet potatoes grown with 1, 2, or 3 t ha^−1^ of broiler litter (Fig. 1). Generally, the ash content of the sweet potatoes increased as the broiler litter rate increased, with a high linear correlation (*r* = 0.90) (Table 4). Although treatment T_2_ sweet potatoes had slightly less ash content than T_1_, the difference was not statistically significant. The increase in ash content of the sweet potatoes as the broiler litter rate increased may have been due to the higher availability of the mineral nutrients in the soil and hence, higher absorption. Sistani et al. (2008) found that higher application of broiler litter resulted in higher accumulation of P, K, Mg, and Ca in the soil. Agbede and Adekiya (2011) also observed that the leaf N, P, and K in sweet potato gradually increased as the level of poultry manure applied was increased from 0, 5, 10 to 15 t ha^−1^. In addition, Demir et al. (2010) found that the P concentration of tomato leaves and fruits significantly increased with increase in poultry manure application rate. Agbede (2010) also found that addition of poultry manure significantly increased the nutrient (N, P, K, Ca, and Mg) concentration in the sweet potatoes leaves. The current findings were consistent with these past studies as it showed that higher rate of application of the broiler litter resulted in higher amounts of ash content.

### Fiber content

The crude fiber contents of the sweet potato treatments had a very narrow range from 0.89% for T_0.5_ to 0.99% for the T_3_ (Table 1). Considering the moisture content of the sweet potatoes (Table 1), the fiber content range was equivalent to 3.9–4.5%, on dry weight basis, which was consistent with findings from some previous studies. Mullin et al. (1994) found the insoluble fiber content of fresh, cured, and stored Beauregard sweet potatoes to be 4.70%, 4.60%, and 5.02%, respectively, on dry weight basis. This observation was consistent with the findings in the present study, because crude fiber mainly accounts for the insoluble fiber (Knudsen 2001). Oboh et al. (1989) also found that the crude fiber content of 49 sweet potato varieties ranged between 3.45% and 6.36% on dry basis. In fact, the two light orange-fleshed sweet potatoes among the 49 varieties had crude fiber contents of 4.00% and 4.42%. In the current study, the broiler litter application rate did not show much effect on the crude fiber content of the Beauregard sweet potato. The fiber contents of all the treatments were not significantly different from that of the control. Only T_0.5_ had statistically slightly lower fiber content than the T_3_ sweet potatoes. However, the fiber content had a moderate positive linear correlation (*r* = 0.47) with the rate of broiler litter application (Table 4).

### Vitamin C content

**Figure 2:**
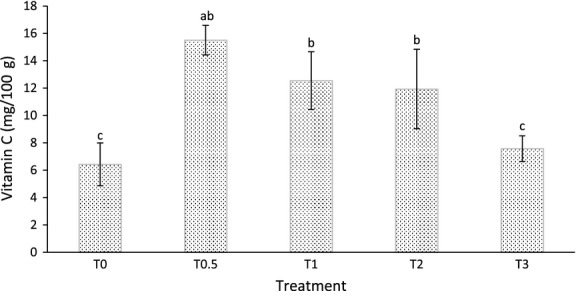
Vitamin C content of the sweet potatoes growth with different amounts of broiler litter.

**Figure 3:**
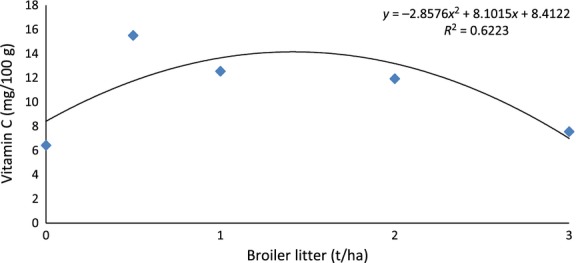
Quadratic relationship between the vitamin C content of the sweet potato and the rate of broiler litter (t ha^−1^) applied.

Treatment T_0_ (6.43 mg/100 g) sweet potatoes had the least ascorbic acid content, whereas T_0.5_ (15.51 mg/100 g) had the highest amount (Fig. 2), a range close to the findings of some past studies. Using Voltammetric and titrimetric methods, Ogunlesi et al. (2010) found the vitamin C of sweet potato to be 6.15 and 4.28 mg/100 g, respectively, and in another study, Watada and Tran (1987) found the content to be 27.7 mg/100 g, on fresh weight basis. In the current study, vitamin C content of the sweet potatoes peaked at T_0.5_ and then gradually declined as the broiler litter rate increased (Fig. 2). In fact, vitamin C content of treatment T_3_ (7.57 mg/100 g) was not statistically different from that of T_0_. The vitamin C content showed no linear correlation with the rate of broiler litter applied (Table 4); however, the two parameters portrayed a fairly strong, concave down, quadratic relationship (*R*^2^ = 0.62) (Fig. 3). Some past studies have also shown that the level/type/method of soil fertilization do affect the vitamin C content with similar trend as observed in the current study. Using five levels of poultry manure (0, 5, 10, 15, 20 t ha^−1^), Ademoyegun et al. (2011) found that the vitamin C content of pepper peaked at 5 t ha^−1^, and then declined as the manure application rate increased. Mathur et al. (2010) also observed that tomato fruits grown with the higher rate of 8 m^3^/20 m^2^ of chicken manure had less vitamin C content than fruits fertilized with the lower rate of 4 m^3^/20 m^2^ of the manure. Xu et al. (2001) observed that soil fertilization method/type do influence vitamin C content of crop. Possibly, elevation of N at higher broiler litter rate is the reason for the quadratic relationship as high concentration have been shown to cause drop in vitamin C content (Lee and Kader 2000).

### *β*-Carotene content

Treatment T_0.5_ sweet potatoes had the highest (263 *μ*g/g) *β*-carotene content, which was significantly higher than all the other treatments, except T_3_ (Table 1). T_1_ (139 *μ*g/g) sweet potatoes had the least *β*-carotene content. However, there was a gradual increase in the *β*-carotene content from T_1_ to T_3_ (238 *μ*g/g) (Table 1). Nedunchezhiyan et al. (2010) found that application of farm yard manure increased *β*-carotene content in sweet potato, compared to the roots which were grown without soil fertilization. Rattler et al. (2005) also observed that *β*-carotene content of lettuce increased significantly with increase in the rate of soil fertilization. In addition, Kipkosgei et al. (2003) working with black nightshade and Naikwade et al. (2011) with spinach also observed that *β*-carotene content was significantly increased depending on the type and the level of fertilizer applied. However, in the current study, there was weak linear relationship (*r* = 0.23) between the *β*-carotene content and the broiler litter (Table 4).

### Texture

**Figure 4:**
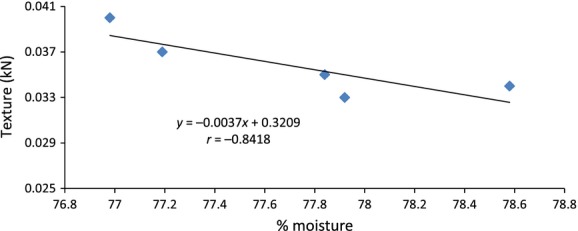
Linear correlation between firmness and moisture content of the sweet potatoes.

The firmness of the sweet potatoes ranged from 0.033 to 0.040 kN, for treatments T_0_ and T_0.5_, respectively (Table 3). Only treatment T_0.5_ had significantly firmer sweet potatoes than the control. Although the firmness of the treatments (T_1_, T_2_, and T_3_) appeared slightly higher than that of the control, they were not significant different (Table 3). T_0.5_ sweet potatoes appeared firmer possibly because they had the least moisture content (Table 1) compared to all the other treatments. The firmness of the sweet potatoes had a very strong negative correlation (*r* = −0.84) to the moisture content (Fig. 4); however, the linear relationship (*r* = −0.20) with the broiler litter rate was very weak (Table 4).

### Color

**Figure 5:**
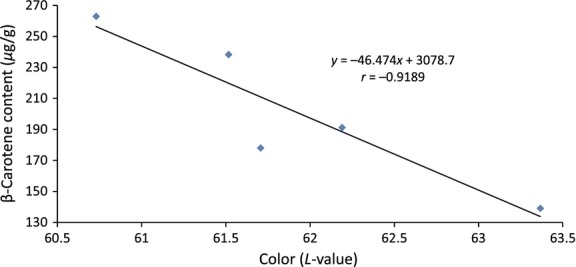
Correlation between the *β*-carotene content and color *L*-value of the sweet potato treatments.

Treatment T_0.5_ sweet potatoes had the darkest (*L* = 60.7) orange color (Table 5), which may have been due to the high *β*-carotene content as reflected in Table 2. On the other hand, sweet potatoes grown with 1 t ha^−1^ of broiler litter which were lightest (*L* = 63.4) in color, had the least amount of *β*-carotene. Generally, deeper orange sweet potatoes had higher *β*-carotene content than the lighter ones as reflected in the correlation between *β*-carotene content and the color *L*-values (Fig. 5). The two characteristics had a negative correlation with *r* = −0.9189. Kidmose et al. (2007) observed that sweet potato with darker orange flesh had higher *β*-carotene content than the yellow-fleshed variety. Ameny and Wilson (1997) also found that the color *L*-value of sweet potatoes analyzed had a negative correlation (*r* = −0.74) to the *β*-carotene content. However, the color b-value which is the measure of yellowness, increased as the broiler litter rate increased, with a strong linear correlation of *r* = 0.8563.

**Table 5:** Color values of the sweet potatoes grown with the different amounts of broiler litter

Treatment	*L*-value	*a*-value	*b*-value
T_0_	61.71 ± 0.18^bc^	20.24 ± 0.20^a^	23.33 ± 0.34^b^
T_0.5_	60.73 ± 0.32^c^	19.85 ± 0.25^a^	22.12 ± 0.47^c^
T_1_	63.37 ± 0.44^a^	20.67 ± 0.23^a^	24.73 ± 0.57^a^
T_2_	62.19 ± 0.049^b^	20.34 ± 0.56^a^	23.32 ± 0.65^b^
T_3_	61.52 ± 1.09^bc^	20.1 ± 0.77^a^	24.71 ± 0.43^a^

Values on the same column with similar letters are not significantly different (*P* < 0.05). T_0_, T_0.5_, T_1_, T_2_, T_3_ refer to treatments and the subscripts represent tons of broiler litter applied.

## Conclusion

Addition/increase in broiler litter affected some of the nutritional properties of the Beauregard sweet potatoes. Ash content of the sweet potatoes increased as the broiler litter application rate was increased, probably due to the accumulation of mineral nutrients, like P, K, Mg, Ca, in the roots. Application of broiler litter at the lowest rate significantly enhanced the vitamin C content in the sweet potatoes. However, the vitamin C content gradual decreased as the broiler litter rate was further increased. Also, the least broiler litter application rate yielded sweet potatoes with the highest *β*-carotene. Although T1 had the least amount of *β*-carotene, the content gradually rose with further increase in broiler litter rate. Moisture, fiber, and texture of the sweet potatoes were not significantly influenced by the broiler litter application. Overall, the sweet potatoes grown with 0.5 t ha^−1^ of broiler litter had higher dry matter, vitamin C, and *β*-carotene contents, and also were firmer with darker orange color than the control. These results show that qualitative Beauregard sweet potatoes can be grown organically using broiler litter application rate as recommended by the Alabama Cooperative Extension Program, minimizing the cost of production and the potential of surface and ground water contamination.
